# High Myeloperoxidase Positive Cell Infiltration in Colorectal Cancer Is an Independent Favorable Prognostic Factor

**DOI:** 10.1371/journal.pone.0064814

**Published:** 2013-05-29

**Authors:** Raoul A. Droeser, Christian Hirt, Serenella Eppenberger-Castori, Inti Zlobec, Carsten T. Viehl, Daniel M. Frey, Christian A. Nebiker, Raffaele Rosso, Markus Zuber, Francesca Amicarella, Giandomenica Iezzi, Giuseppe Sconocchia, Michael Heberer, Alessandro Lugli, Luigi Tornillo, Daniel Oertli, Luigi Terracciano, Giulio C. Spagnoli

**Affiliations:** 1 Department of Surgery, University Hospital Basel, Switzerland; 2 Institute of Surgical Research and Hospital Management (ICFS) and Department of Biomedicine, University of Basel, Switzerland; 3 Institute of Pathology, University Hospital Basel, Switzerland; 4 Institute for Pathology, University of Bern, Switzerland; 5 Institute of Translational Pharmacology, National Research Council, Rome, Italy; 6 Department of Surgery, Ospedale Regionale di Lugano, Switzerland; 7 Department of Surgery, Cantonal Hospital Olten, Switzerland; Health Canada, Canada

## Abstract

**Background:**

Colorectal cancer (CRC) infiltration by adaptive immune system cells correlates with favorable prognosis. The role of the innate immune system is still debated. Here we addressed the prognostic impact of CRC infiltration by neutrophil granulocytes (NG).

**Methods:**

A TMA including healthy mucosa and clinically annotated CRC specimens (n = 1491) was stained with MPO and CD15 specific antibodies. MPO+ and CD15+ positive immune cells were counted by three independent observers. Phenotypic profiles of CRC infiltrating MPO+ and CD15+ cells were validated by flow cytometry on cell suspensions derived from enzymatically digested surgical specimens. Survival analysis was performed by splitting randomized data in training and validation subsets.

**Results:**

MPO+ and CD15+ cell infiltration were significantly correlated (p<0.0001; r = 0.76). However, only high density of MPO+ cell infiltration was associated with significantly improved survival in training (*P* = 0.038) and validation (*P* = 0.002) sets. In multivariate analysis including T and N stage, vascular invasion, tumor border configuration and microsatellite instability status, MPO+ cell infiltration proved an independent prognostic marker overall (*P* = 0.004; HR = 0.65; CI:±0.15) and in both training (*P* = 0.048) and validation (*P* = 0.036) sets. Flow-cytometry analysis of CRC cell suspensions derived from clinical specimens showed that while MPO+ cells were largely CD15+/CD66b+, sizeable percentages of CD15+ and CD66b+ cells were MPO−.

**Conclusions:**

High density MPO+ cell infiltration is a novel independent favorable prognostic factor in CRC.

## Introduction

Outgrowth and progression of human colorectal cancers (CRC) are driven by gene mutations and microsatellite instability tumor inherent characteristics [1], [2], and by the interaction of cancer cells with microenvironmental stimuli provided by non-transformed cells [3], [4]. In particular, cytokine and chemokine environment and infiltration by immunocompetent cells significantly influence CRC outcome [5]–[8].

Infiltration by activated CD8+ memory T cells and expression of IFN-γ gene within CRC were convincingly shown to be associated with favorable prognosis [5], [7]. Furthermore, we and others have shown that FOXP3+ immune cell infiltration independently predicts improved survival in CRC [9], [10].

The role of innate immune system cells was not studied in comparable detail and controversial data were reported regarding CRC infiltration by NK cells [11]–[14] and macrophages [15]–[17].

Granulocytes have largely been disregarded by tumor immunologists [18]. However, recent studies, mainly performed in experimental models, suggest that neutrophil granulocytes might prevent metastatic cancer progression [19]. Furthermore, they were suggested to undergo cytokine driven differentiation into N1 and N2 cells endowed with anti- and pro-tumor properties, respectively [20], [21]. These findings have led to a resurgent interest in granulocyte infiltration in cancer [22]–[24].

In previous work, we showed that CRC infiltration by CD33+/HLA-DR−/CD16+ myeloid cells is associated with improved patient survival [13]. Based on these phenotypic features, we hypothesized that CRC could be infiltrated by granulocytes with a favorable prognostic significance.

Myeloperoxidase (MPO) is a lysosomal enzyme produced in high amounts by neutrophilic granulocytes (NG) [25], especially during their early maturation phase. MPO catalyzes the production of hypochlorous acid from hydrogen peroxide and chloride anion and oxidizes tyrosine to tyrosyl radicals. Both hypochlorous acid and tyrosyl radicals are cytotoxic to a variety of microorganisms. Notably, MPO is also involved in the induction of granulocyte apoptosis following activation [26], [27].

In a small series of CRC samples (n = 67), it has been shown that MPO+ cell infiltration is significantly higher in CRC than in normal colon mucosa [28]. However, prognostic relevance of CRC infiltration by MPO+ cells has not been addressed so far.

CD15, also known as Lewis X and stage-specific embryonic antigen 1, is a carbohydrate adhesion molecule expressed on mature neutrophils, mediating phagocytosis and chemotaxis [29]. Importantly, CD15 expression has been detected in tumor cells and found to correlate with poor prognosis in head and neck, gastric and lung cancers [30]–[32]. In CRC, expression of CD15 on tumor cells was shown to occur during progression to metastatic stages [33] and to be associated with high incidence of recurrences and poor survival [34], [35]. However, the prognostic value of CRC infiltration by CD15+ immune cells has not been explored.

Here we show for the first time that a subgroup of CRC is characterized by a high infiltration by MPO+ and CD15+ positive cells. Most importantly, high MPO+ cell density in CRC is independently associated with favorable prognosis.

## Materials and Methods

### Ethics Statement

Written consent has been given from the patients for their information to be stored in the hospital database and used for research. The use of this clinically annotated TMA for research was approved by the corresponding Ethics Committee of the University Hospital of Basel (Ethikkommission beider Basel) and the ex vivo analyses were approved by the Institutional Review Board (63/07). For freshly excised clinical specimens included in this study written consent has been given from the patients undergoing surgical treatment at Basel University Hospital.

### Tissue Microarray Construction

The TMA used in this work was constructed by using 1420 non-consecutive, primary CRCs, as previously described [36]. Briefly, formalin-fixed, paraffin-embedded CRC tissue blocks were obtained. Tissue cylinders with a diameter of 0.6 mm were punched from morphologically representative areas of each donor block and brought into one recipient paraffin block (30×25 mm), using a semiautomated tissue arrayer. Each punch was made from the center of the tumor so that each TMA spot consisted of at least a 50% of tumor cells. One core per case was used.

### Clinicopathological Features

Clinicopathological data for the 1420 CRC patients included in the TMA were collected retrospectively in a non-stratified and non-matched manner. Annotation included patient age, tumor diameter, location, pT/pN stage, grade, histologic subtype, vascular invasion, border configuration, presence of peritumoral lymphocytic inflammation at the invasive tumor front and disease-specific survival (table 1). Tumor border configuration and peritumoral lymphocytic inflammation were evaluated according to Jass using the original H&E slides of the resection specimens corresponding to each tissue microarray punch [37]. The number of lymph nodes evaluated ranged between 1 and 61 with mean and median of 12 and 11, respectively. MMR status was evaluated by IHC according to MLH1, MSH2 and MSH6 expression [38]. Based on this analysis, the TMA included 1031 MMR-proficient tumors and 194 MMR-deficient tumors.

**Table 1:** Characteristics of CRC patient cohort (n = 1420)*.

Characteristics	Number of cases or mean	Percentage or range
**Age, years**	71	(30–96)
**Tumor size (mm)**	75	(4–170)
**Gender**		
**Female (%)**	741	(52.2)
**Male (%)**	673	(47.4)
**Anatomic site of the tumor**		
**Left-sided (%)**	912	(64.2)
**Right-sided (%)**	488	(34.4)
**T stage**		
**T1 (%)**	62	(4.4)
**T2 (%)**	203	(14.3)
**T3 (%)**	899	(63.3)
**T4 (%)**	223	(15.7)
**N stage**		
**N0 (%)**	711	(50.1)
**N1 (%)**	358	(25.2)
**N2 (%)**	294	(20.7)
**Tumor grade**		
**G1 (%)**	31	(2.2)
**G2 (%)**	1177	(82.9)
**G3 (%)**	177	(12.5)
**UICC**		
**Stage I (pN0 pT1 or 2) (%)**	185	(13.6)
**Stage IIA (pN0 pT3)+IIB-C (pN0 pT4) (%)**	445+61	(37.2)
**Stage III (pN>0) (%)**	581	(42.7)
**Stage IV metastasis (%)**	88	(6.5)
**Tumor border configuration**		
**Infiltrative (%)**	871	(61.3)
**Pushing (%)**	513	(36.1)
**Vascular invasion**		
**No (%)**	1002	(70.6)
**Yes (%)**	383	(27)
**Microsatellite Stability**		
**Proficient (%)**	1031	(72.6)
**Deficient (%)**	194	(13.7)
**Rectal cancers (%)**	575	(40.5)
**Overall survival time (months)**	67.7	(0–152)
**5-years survival % (95%CI)**	56.4	(54–59)

* percentage may not add to 100% due to missing values of same variables; age and tumor size were evaluated using the Kruskal-Wallis test. Gender, anatomical site, T stage, N stage, grade, vascular invasion, and tumor border configuration were analyzed using the χ^2^ test. Survival analysis was performed using the Kaplan-Meier method.

Overall survival was defined as primary endpoint. Follow-up data were available for 1379 patients with mean/median and IQR event-free follow-up time of 67.7/68 and 45–97 months.

### Immunohistochemistry

Standard indirect immunoperoxidase procedures were used for immunohistochemistry (IHC; ABC-Elite, Vector Laboratories, Burlingame, CA). Briefly, slides were dewaxed and rehydrated in distilled water. Endogenous peroxidase activity was blocked using 0.5% H2O2. Sections were incubated with 10% normal goat serum (DakoCytomation, Carpinteria, CA) for 20 min and incubated with primary antibody at room temperature. Primary antibodies used were specific for MPO (clone 59A5 Novocastra, Newcastle, UK), CD15 (clone Carb-1, Leica Biosystems, Nussloch, Germany), CD16 (clone 2H7, Novocastra), CD68 (clone PG-M1, Dako, Glostrup, Denmark), FOXP3 (clone 236A/E7, Abcam, Cambridge, UK) and CD8 (clone C8/144B, DakoCytomation, Switzerland). Subsequently, sections were incubated with peroxidase-labelled secondary antibody (DakoCytomation) for 30 min at room temperature. For visualization of the antigen, sections were immersed in 3-amino-9-ethylcarbazole plus substrate-chromogen (DakoCytomation) for 30 min, and counterstained with Gill’s hematoxylin.

### Evaluation of Immunohistochemistry

MPO+ and CD15+ tumor infiltrating cells were counted for each punch (approximately one high power [20x] field) by a trained research fellow [R.D.]. Data were independently validated by two additional investigators [L.To. and C.H.] and a high Spearman correlation coefficient ( = 0.82) and a highly significant (p<0.0001) correlation between measurements was observed. Evaluation of MLH1, MSH2, MSH6, CD16, CD68, CD8 and FOXP3 specific stainings in the CRC TMA under investigation was published previously [9], [13], [39].

### Flow Cytometry Analyses

Following Institutional Review Board approval (63/07), tissues from surgically removed CRC and adjacent normal mucosa were minced, centrifuged, and resuspended in RPMI 1640 medium supplemented with 5% foetal calf serum, 2 mg/ml collagenase IV, 0.1 mg/ml hyaluronidase V, and 0.2 mg/ml DNAse I (Sigma Aldrich, Basel, Switzerland). Following a 1 hour digestion, cell suspensions were filtered and centrifuged. For phenotypic analysis of surface markers, cells were stained with mAbs for 15 minutes on ice in PBS, washed once with PBS 0.5% FCS, 0.5 M EDTA buffer and fixed in lysis buffer from BD Bioscience (1∶10). Samples were then permeabilized in BD fixation/permeabilization buffer. For intracellular staining, an anti-MPO reagent or an isotype matched negative control antibody were added for a 15 min incubation at room temperature. After a PBS wash, cells were suspended in wash buffer and analyzed by flow cytometry using a 2-laser BD FACSCalibur (Becton Dickinson, San Jose, CA). Results were analyzed by Cell Quest (Becton Dickinson, San Jose, CA) and Flow Jo (Tree Star, Ashland, OR) softwares.

### Statistical Analysis

Cut-off scores used to classify CRC with low or high MPO+ or CD15+ infiltration were obtained by regression tree analysis, evaluating sensitivity and false positive rate for the discrimination of survivors and non-survivors, on all tumor samples [40]. Specific scores were set at 60 cells/TMA-punch for MPO+ and at 46 cells/TMA-punch for CD15+ infiltration. Chi-Square or Fisher’s Exact tests were used to determine the association of MPO+ and CD15+ infiltration and clinicopathological features. Univariate survival analysis was carried out by the Kaplan-Meier method and log rank test. The assumption of proportional hazards was verified for both markers by analyzing the correlation of Schoenfeld residuals and the ranks of individual failure times. Any missing clinicopathological information was assumed to be missing at random. Subsequently, MPO+ and CD15+ cell infiltration data were entered into multivariate Cox regression analysis and hazard ratios (HR) and 95% confidence intervals (CI) were used to determine associations with survival time. The multivariate Cox regression analysis was performed with 975 cases since missing values were excluded from the model.

Spearman’s rank correlation was used to analyze the correlation between MPO+, CD15+, CD16+, CD68+, CD8+ and FOXP3+ cell infiltration. Statistical analyses were performed using R i386 Version 2.15.2 (http://www.R-project.org). Data reporting was performed according to the REMARK criteria [41].

## Results

### CRC Infiltration by MPO+ and CD15+ Cells: Detection and Association with Clinicopathological Features

**Figure 1:**
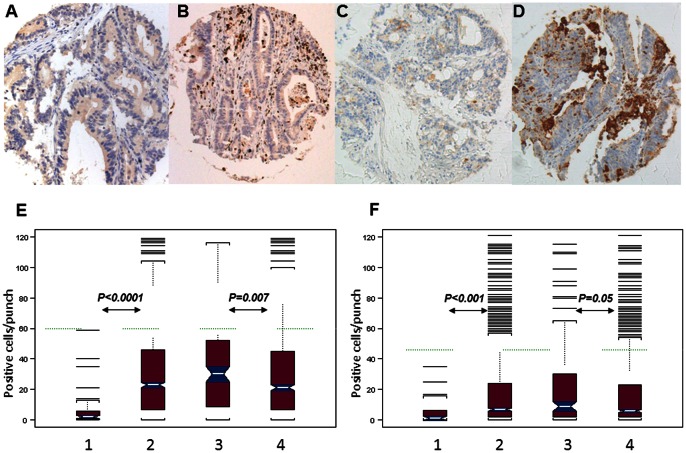
MPO and CD15 specific staining in CRC. CRC samples were stained with MPO and CD15 specific monoclonal antibodies (clone 59A5, Novocastra and clone Carb-1, Leica, respectively). Tumor punches are representative of low (panels A and C) and high (panels B and D) density of CRC infiltrating MPO+ (panels A–B) and CD15+ (panels C–D) cells, respectively. Magnification: 20x. Panel E reports the distribution of MPO+ cells in normal mucosa (E1), total CRC (E2), MMR-deficient CRC (E3) and MMR-proficient CRC (E4). The green line indicates the cut-off of 60 cells/punch as defined by regression tree analysis. Panel F reports the distribution of CD15+ cells in normal mucosa (F1), total CRC (F2), MMR-deficient CRC (F3) and MMR-proficient CRC (F4). The green line indicates the cut-off of 46 cells/punch as defined by regression tree analysis.

MPO+ and CD15+ cells could be successfully identified in the TMA under investigation by specific mAbs. Figure 1A–D shows representative stainings of CRC with low and high MPO+ or CD15+ cell infiltration.

Out of 1420 CRC individual specimens, MPO expression could be evaluated in 1225 samples including 1031 MMR-proficient and 194 MMR-deficient CRC. CD15 expression was evaluable in 1191 CRC specimens, including 817 MMR proficient and 191 MMR deficient samples. Numbers of samples evaluated for each feature are indicated in absolute numbers and percentages in table 2. Dropouts were due to loss of punches during TMA staining preparation or missing information, and usually accounted for <15% of data (tables 1–2).

**Table 2:** Association of MPO+ and CD15+ low and high immune cell density with clinicopathological features in CRC.

		MPO−		MPO+		p-value	CD15 –		CD15+		p-value
		N = 1047	(85.4%)	N = 178	(14.5%)		N = 1062	(89.2%)	N = 129	(10.8%)	
**Age**	years	71	(30–96)	73	(37–96)	**0.04**	71	(30–96)	71	(38–96)	0.45
**Tumor diameter**	mm	49	(8–120)	45	(4–170)	0.84	45	(4–160)	45	(8–170)	0.99
**Gender**	Female	546	(52.1)	90	(50.6)		558	(52)	70	(54.3)	0.64
	Male	501	(47.9)	88	(49.4)	0.75	515	(48)	59	(45.7)	
**Tumor location**	Left-sided	674	(64.4)	117	(65.7)	0.86	677	(63.1)	95	(73.6)	**0.018**
	Right-sided	361	(34.5)	60	(33.7)		385	(35.9)	32	(24.8)	
**Histologic subtype**	Mucinous	86	(8.2)	16	(9)	0.66	97	(9)	7	(5.4)	0.19
	Non-mucinous	955	(91.2)	158	(88.8)		965	(89.9)	122	(94.6)	
**pT stage**	pT1–2	182	(17.4)	48	(27)	**0.007**	190	(17.7)	41	(31.8)	**0.0004**
	pT3–4	833	(79.6)	129	(72.5)		857	(79.9)	87	(67.4)	
**pN stage**	pN0	527	(50.3)	101	(56.7)	0.16	536	(50)	72	(55.8)	0.35
	pN1-2	479	(45.7)	72	(40.4)		494	(46)	55	(42.6)	
**Tumor grade**	G1-2	894	(85.4)	146	(82)	0.224	907	(84.5)	110	(85.3)	0.68
	G3	129	(12.3)	28	(15.7)		138	(12.9)	18	(14)	
**Vascular invasion**	Absent	740	(70.7)	133	(74.7)	0.27	754	(70.3)	102	(79.1)	0.07
	Present	283	(27)	41	(23)		291	(27.1)	26	(20.2)	
**Tumor border**	Pushing	385	(36.8)	75	(42.1)	0.178	404	(37.7)	45	(34.9)	0.44
	Infiltrating	637	(60.8)	99	(55.6)		640	(59.6)	83	(64.3)	
**PTL inflammation**	Absent	819	(78.2)	132	(74.2)	0.224	835	(77.8)	97	(75.2)	0.3
	Present	205	(19.6)	42	(23.6)		211	(19.7)	31	(24)	
**Local recurrence**	Absent	212	(20.2)	54	(30.3)	**0.031**	241	(22.5)	26	(20.2)	**0.03**
	Present	162	(15.5)	23	(12.9)		180	(16.8)	8	(6.2)	
**Distant metastasis**	Absent	304	(29)	69	(38.8)	0.108	343	(32)	32	(24.8)	0.08
	Present	75	(7.2)	9	(5.1)		84	(7.8)	2	(1.6)	
**Microsatellite stability**	Deficient	159	(15.2)	35	(19.7)	0.161	167	(15.6)	24	(18.6)	0.45
	Proficient	888	(86.1)	143	(13.9)		729	(67.9)	88	(68.2)	
**5-year survival rate**	(95%CI)	55.6	(52.4–59)	68.5	(61.3–76.5)	**0.0009**	56.8	(53.6–60.2)	63.6	(55.1–73.4)	**0.033**

* percentage may not add to 100% due to missing values of same variables; variables are indicated as absolute numbers, %, median or range; age and tumor size were evaluated using the Kruskal-Wallis test. Gender, anatomical site, T stage, N stage, grade, vascular invasion, and tumor border configuration were analyzed using the χ^2^ test. Survival analysis was performed using the Kaplan-Meier method.

Normal colonic mucosa was indeed infiltrated by MPO+ and CD15+ cells (mean: 6.4, median: 2, range 0–59 cells/punch for MPO (n = 48) and mean: 4.3, median: 1, range 0–35 cells/punch for CD15 (n = 47), respectively). However, a significantly higher (*P*<0.001) infiltration by MPO+ and CD15+ cells was detectable in CRC samples (mean: 26.7, median: 23, range 0–150 cells/punch for MPO (n = 1225) and mean: 16.4, median: 7, range 0–125 cells/punch for CD15 (n = 1191), respectively; figure 1E–F).

**Figure 2:**
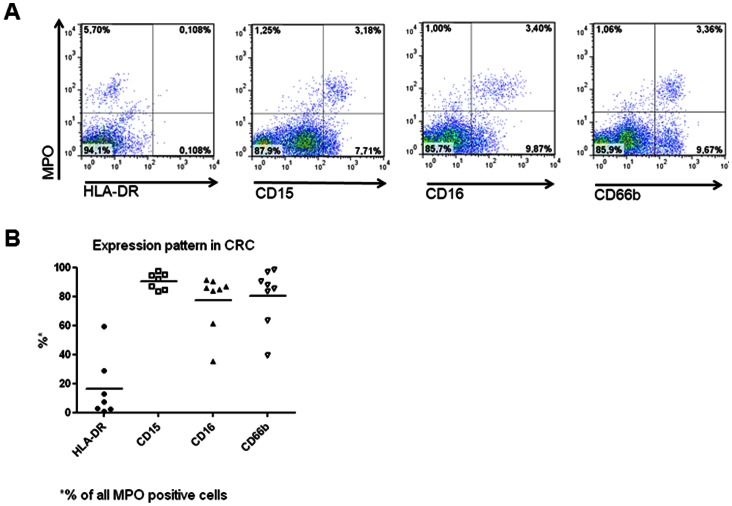
Phenotypic characterization of CRC infiltrating MPO+ cells. CRC surgical specimens were enzymatically digested and immediately stained with fluorochrome labeled mAbs recognizing MPO, HLA-DR, CD66b, CD15 and CD16, as indicated in “materials and methods”. Panel A reports one representative staining, whereas panel B summarizes results from freshly excised specimens (n = 8) regarding the expression of the indicated markers in CRC infiltrating MPO+ cells.

MPO+ and CD15+ infiltration, as evaluated by absolute cell numbers, was significantly higher in MMR deficient than in MMR proficient CRC (median: 30 cells/punch in deficient vs. 21 cells/punch in proficient CRC *P* = 0.007 and 9 cells/punch in deficient vs 6 cells/punch in proficient CRC *P* = 0.05, for MPO and CD15 respectively; figures 2E–F).

Regression tree analysis defined cut-off scores for MPO+ and CD15+ CRC infiltrating cells detected in individual punch biopsies (n = 60 and n = 46, respectively) were used to assess clinicopathological correlations.

Univariate Cox regression analysis revealed that high density MPO+ cell infiltration (≥60 cells/punch), detectable in 14.5% of tumors, was significantly associated with older age of patients (*P* = 0.04). Most importantly, it was significantly associated with early (pT1–2) tumor stage (*P* = 0.007), absence of local recurrence (*P* = 0.031) and higher 5-year survival rate (*P* = 0.0009) (table 2).

High density of CD15+ infiltrating cells (≥46 cells/punch), detectable in 10.8% of tumors, was significantly associated with left sided location (*P* = 0.018), early (pT1–2) stage (*P* = 0.0004), absence of local recurrence (*P* = 0.03) and higher 5-year survival rate (*P* = 0.033) (table 2).

### Correlation between MPO+, CD15+, CD16+ and CD68+ Tumor-infiltrating Myeloid Cells

To explore relationships between tumor infiltration by cells expressing MPO and other immune markers (CD15, CD16, CD68, CD8, FOXP3) expressed by immunocompetent cells infiltrating CRC, data from additional immune-histochemical stainings of the same TMA from previous studies were used [9], [13], [39]. The statistically strongest correlation was detectable between MPO+ and CD15+ cell infiltration (r = 0.75, *P*<0.0001), whereas correlations of lesser significance were detectable with CD16+ (r = 0.32), CD68+ (r = 0.35), CD8+ (r = 0.13) and FOXP3+ (r = 0.21) cell infiltration.

### Ex vivo Characterization of MPO+ cells from Freshly Removed CRC

To assess in detail phenotypic characteristics of tissue infiltrating MPO+ cells, freshly excised CRC (n = 8) were enzymatically digested, and single cell suspensions were analyzed by flow cytometry. Examples of this phenotypic analysis are reported in figure 2a.

The large majority of CRC infiltrating, MPO+ cells were CD15+ (90.3%±5.6%), CD16+, (77.6±19.4%), and CD66b+ (80.6±19.9%; figure 2b). Interestingly, MPO+ cells detectable in autologous normal mucosa displayed a similar (P>0.05) marker expression pattern (data not shown). Notably however, sizeable percentages of MPO−/CD66b+ cells (47.3±35.7%) and, more expectedly, of MPO−/CD15+ cells (78.0±17.7%) of possibly cancerous nature were also detectable in CRC derived cell suspensions.

### Prognostic Significance of MPO+ and CD15+ Cell Infiltration in the CRC Microenvironment

**Figure 3:**
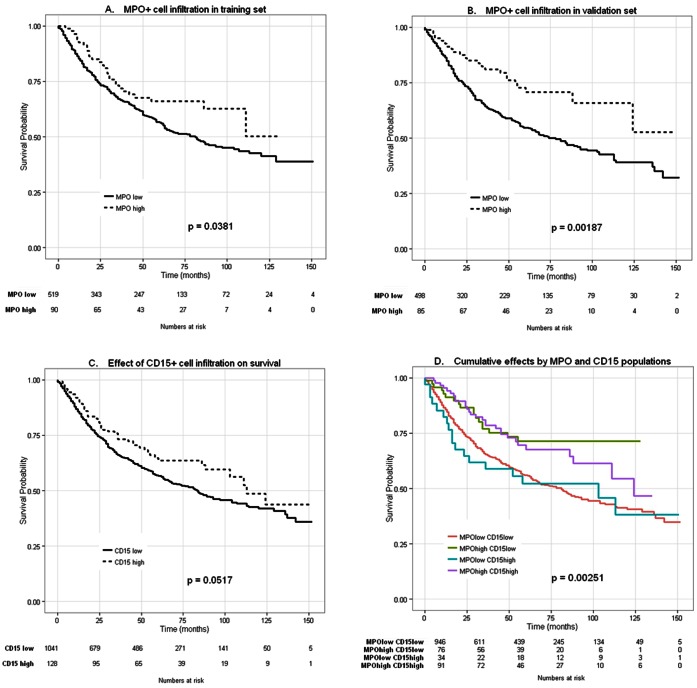
Effects of MPO+ and CD15+ tumor infiltration on overall survival in patients with CRC. Kaplan-Meier overall survival curves were designed according to MPO+ and CD15+ tumor infiltration in patients bearing CRC. In panels A–C, dotted lines refer to high density and black lines to low density infiltration according to cut-off values established by regression tree analysis (60 cells/punch for MPO+ and 46 cells/punch for CD15+ cell infiltration). Panels A and B report the effects of high MPO+ cell infiltration, as detected in the training (n = 609; 255 deaths observed in 519 patients with low CRC infiltration by MPO+ cells and 28 deaths observed in 90 patients with tumors with high MPO+ cell infiltration, *P* = 0.038) and in the validation set (n = 583; 234 deaths observed in 498 patients with low CRC infiltration by MPO+ cells and 23 deaths observed in 85 patients with tumors with high MPO+ cell infiltration, *P* = 0.002). Panel C reports the effect of high CD15+ CRC infiltration in the whole group of patients under investigation (n = 1169; 458 deaths observed in 1041 patients with low CRC infiltration by CD15+ cells and 47 deaths observed in 128 patients with tumors with high CD15+ cell infiltration, *P* = 0.051). In panel D cumulative effects of tumor infiltration by MPO+ and CD15+ cells were explored. Rosa line (430/946) refers to tumors with low MPO+/CD15+ cell infiltration. Light blue line (18/34) refers to tumors with low MPO+ and high CD15+ cell infiltration. Lila line (29/91) refers to tumors with high MPO+/CD15+ cell infiltration and green line (18/76) refers to CRC with high MPO+ and low CD15+ cell infiltration (n = 1147; 430 deaths observed in 946 patients with low CRC infiltration by MPO+ and CD15+ cells; 18 deaths observed in 34 patients with tumors with high CD15+ and low MPO+ cell infiltration; 29 deaths observed in 91 patients with high CRC infiltration by MPO+ and CD15+ cells; 18 deaths observed in 76 patients with tumors with high MPO+ and low CD15+ cell infiltration, *P* = 0.002).

Median survival time was 50 and 46 months for patients with high or low MPO+ cell density, respectively. High MPO+ cell infiltration was significantly (P = 0.0003) associated with better prognosis (0.59 HR, 95%CI: 0.45–0.74), as compared to tumors with low MPO+ cell infiltration in univariate Cox regression analysis. Upon splitting of the cohort in a test and a validation set, high score MPO+ cell infiltration was still associated with significantly improved survival (*P* = 0.038 and *P* = 0.002, respectively; figure 3A–B). Several randomizations of the overall cohort were tried and all results were found to be comparable.

In univariate analysis survival was also increased in case of high score CD15+ cell infiltration (*P* = 0.051, figure 3C). A combination analysis however, showed that MPO is the dominant marker associated with improved prognosis, without relevant additive benefit provided by CD15 positivity (figure 3D).

Most importantly, in multivariate analysis, high score MPO+, but not CD15+, cell infiltration was independently associated with favorable prognosis after adjusting for several known prognostic factors such as age, sex, T stage, N stage, tumor grade, vascular invasion, tumor border configuration and microsatellite stability (*P* = 0.004; table 3). Also in the two stratified collectives the effect of MPO+ cell infiltration on survival of patients with CRC remained significant (*P* = 0.048 and *P* = 0.036 in the testing and validation set, respectively).

**Table 3:** Multivariate Hazard Cox regression survival analysis.

	HR (95% CI)	P-Values
**MPO (low vs high)**	**0.65** (0.5–0.8)	**0.004**
**Age (continuous)**	**1.03** (1.02–1.03)	**<0.0001**
**Gender (men vs women)**	**1.21** (1.16–1.26)	**<0.0001**
**pT stage (1,2,3,4)**	**1.79** (1.71–1.87)	**<0.00001**
**pN stage (0,1,2)**	**1.93** (1.87–1.99)	**<0.0001**
**Tumor Grade (1,2,3)**	**1.23** (1.10–1.36)	**0.11**
**Vascular invasion (0,1)** *	**1.43** (1.33–1.53)	**0.0006**
**Tumor border configuration (0,1)** **	**1.52** (1.41–1.63)	**0.0003**
**Microsatellite stability (deficient vs. proficient)**	**1.70** (1.56–1.84)	**0.0002**

Multivariate analyses showing Hazard Ratios and p-value for all CRC (n = 975, due to missing values, see “materials and methods”) conferred by high MPO density, age, sex, tumor size, nodal status, tumor grade, vascular invasion, tumor border configuration and microsatellite stability.

* 0: absent, 1: present.

** 0: pushing, 1: infiltrating.

## Discussion

To the best of our knowledge, this is the first study identifying MPO+ neutrophil granulocyte tumor infiltration as an independent favorable prognostic factor in CRC.

Myeloid cell infiltration is known to promote tumor growth and to be associated with poor prognosis in a variety of human cancers [42]. In particular, tumor associated macrophages have been indicated as obligate partners for tumor progression and metastasis formation [43]. Granulocyte infiltration has also been found to be associated with poor prognosis in different tumors including lung cancers and renal and hepatocellular carcinoma [44]–[47]. In this context, CRC might represent an interesting exception [48], [49]. Indeed, controversial data have been reported on the prognostic significance of macrophage infiltration in CRC [50]–[54].

Neutrophil infiltration has been found to be increased in the transition from normal to dysplastic and cancerous mucosa [55]. Furthermore, CRC infiltration by CD66b+ cells has recently been proposed to be associated with adverse prognosis [56].

In previous work we showed that CRC infiltration by CD16+ cells correlates with improved survival [13]. This marker is expressed in neutrophils, in a subset of macrophages and in NK cells [57], [58]. Indeed, NK cell infiltration in CRC is negligible [13], [14] and devoid of prognostic significance [59]. Since CRC infiltrating CD16+ cells are also CD11b+/CD33+/HLA-DR-, in this study we focused on the analysis of cells expressing MPO and CD15 granulocyte markers.

In univariate analysis high density CRC infiltration by cells expressing either marker was associated with improved survival. However, following adjustment for multiple comparisons carried out to compensate for the exploratory nature of this analysis this favorable prognostic relevance was maintained for MPO+ but not for CD15+ cell infiltration.

Importantly, in accord with a previously published report [55] we observed that MPO+ and CD15+ cells preferentially colonized CRC tissues while they poorly infiltrated normal colon mucosa, suggesting that they might be specifically recruited to the tumor microenvironment. Interestingly, MPO+ cell infiltration was higher in MMR-deficient than in MMR-proficient CRC as previously suggested by a study conducted with 67 samples from a limited number of cancers (n = 35) [28].

Flow-cytometric analysis of digested paired normal and cancerous tissues indicates that in both healthy mucosa and CRC infiltrating MPO+ cells are CD15+/CD16+/CD66b+/HLA-DR-, consistent with a granulocytic lineage. Most importantly however, we observed substantial percentages of CD66b+/MPO- cells infiltrating CRC. These data might explain the discrepancies between our findings and a previous report focusing on CD66b+ CRC infiltrating cells [56]. Notably, neutrophils with similar phenotypic characteristics have also been found to infiltrate head and neck cancers [60].

Similarly, the presence of CD15+/MPO− cells in healthy mucosa and cancerous tissues might explain the differential prognostic relevance of these markers. Notably however, MPO gene expression was undetectable in CRC or normal mucosa specimens (data not shown) consistent with a mature granulocyte nature of MPO+ infiltrating cells [61].

MPO activity has been suggested to contribute to the pathogenesis of degenerative diseases, including atherosclerosis, multiple sclerosis and Alzheimer disease [62]. Furthermore, high MPO activity or MPO+ cell infiltration have been detected in esophageal [63], and gynecological cancers [64], [65] and in CRC [28], [66], [67], but their prognostic impact was not analyzed.

Despite early reports documenting their ability to mediate tumor cell cytotoxicity [68] granulocytes have largely been neglected by tumor immunologists [18]. However experimental models in the past have indicated that colorectal cancer cells transfected with G-CSF gene can be rejected by tumor infiltrating neutrophils upon interaction with IFN-γ producing T cells [69]. Granulocytes were also shown to express co-stimulatory molecules and to be able to present antigens [70], [71], thus suggesting the possibility of a role in the initiation of antigen specific antitumor responses.

More recently, evidence of the ability of granulocytes to inhibit lung metastasis formation in an experimental breast cancer model was provided [19]. Furthermore, the capacity of granulocytes to undergo TGF-β dependent polarization into N1 and N2 functional profiles, characterized by anti- or pro-tumoral properties, respectively, similarly to macrophages, has also been documented [20].

The molecular background underlying CRC infiltration by MPO+ cells and its prognostic significance is unclear. These phenomena could reflect chemokine production by activated T cells, and therefore indirectly result from ongoing antitumoral adaptive responses. Alternatively, they might be related to the production of granulocyte attracting chemokines by tumor cells. At least CXCL8 (IL-8) is known to be produced by CRC cells. However, its production was suggested to be associated with increased angiogenesis and tumor dissemination [72]. On the other hand, we and others have previously shown that GM-CSF, promoting granulocyte maturation and survival, can also be produced by CRC cells [73], [74].

Our results contribute to the characterization of the complex features inherent in gut microenvironment and with CRC-immune system interaction [75]. Further research is warranted to clarify molecular mechanisms underlying the independent prognostic impact of MPO+ cells in CRC. Importantly, here we show that CRC infiltrating MPO+ cells express CD16 Fcγ intermediate affinity receptor. The ability of granulocytes to mediate antibody dependent cellular cytotoxicity (ADCC) is debated. However, the availability of novel therapeutic mAb with glycoengineered Fc fragments characterized by increased affinity for Fcγ receptors [76] might lead to a reevaluation of the effector significance of granulocytes.

Within this framework, it is tempting to speculate that neutrophil infiltration should be included in current prognostic models for CRC [77] and that it might represent an important novel stratification factor for randomization in specific clinical trials.

## References

[1] Boland CR, Goel A (2010) Microsatellite instability in colorectal cancer. Gastroenterology 138: 2073–2087. 20420947 10.1053/j.gastro.2009.12.064 PMC3037515

[2] Wood LD, Parsons DW, Jones S, Lin J, Sjoblom T, et al (2007) The genomic landscapes of human breast and colorectal cancers. Science 318: 1108–1113. 17932254 10.1126/science.1145720

[3] Coussens LM, Werb Z (2002) Inflammation and cancer. Nature 420: 860–867. 12490959 10.1038/nature01322 PMC2803035

[4] Hanahan D, Weinberg RA (2011) Hallmarks of cancer: the next generation. Cell 144: 646–674. 21376230 10.1016/j.cell.2011.02.013

[5] Galon J, Costes A, Sanchez-Cabo F, Kirilovsky A, Mlecnik B, et al (2006) Type, density, and location of immune cells within human colorectal tumors predict clinical outcome. Science 313: 1960–1964. 17008531 10.1126/science.1129139

[6] Mlecnik B, Tosolini M, Charoentong P, Kirilovsky A, Bindea G, et al (2010) Biomolecular network reconstruction identifies T-cell homing factors associated with survival in colorectal cancer. Gastroenterology 138: 1429–1440. 19909745 10.1053/j.gastro.2009.10.057

[7] Pages F, Berger A, Camus M, Sanchez-Cabo F, Costes A, et al (2005) Effector memory T cells, early metastasis, and survival in colorectal cancer. N Engl J Med 353: 2654–2666. 16371631 10.1056/NEJMoa051424

[8] Tosolini M, Kirilovsky A, Mlecnik B, Fredriksen T, Mauger S, et al (2011) Clinical impact of different classes of infiltrating T cytotoxic and helper cells (Th1, th2, treg, th17) in patients with colorectal cancer. Cancer Res 71: 1263–1271. 21303976 10.1158/0008-5472.CAN-10-2907

[9] Frey DM, Droeser RA, Viehl CT, Zlobec I, Lugli A, et al (2010) High frequency of tumor-infiltrating FOXP3(+) regulatory T cells predicts improved survival in mismatch repair-proficient colorectal cancer patients. Int J Cancer 126: 2635–2643. 19856313 10.1002/ijc.24989

[10] Salama P, Phillips M, Grieu F, Morris M, Zeps N, et al (2009) Tumor-infiltrating FOXP3+ T regulatory cells show strong prognostic significance in colorectal cancer. J Clin Oncol 27: 186–192. 19064967 10.1200/JCO.2008.18.7229

[11] Coca S, Perez-Piqueras J, Martinez D, Colmenarejo A, Saez MA, et al (1997) The prognostic significance of intratumoral natural killer cells in patients with colorectal carcinoma. Cancer 79: 2320–2328. 9191519 10.1002/(sici)1097-0142(19970615)79:12<2320::aid-cncr5>3.0.co;2-p

[12] Halama N, Braun M, Kahlert C, Spille A, Quack C, et al (2011) Natural killer cells are scarce in colorectal carcinoma tissue despite high levels of chemokines and cytokines. Clin Cancer Res 17: 678–689. 21325295 10.1158/1078-0432.CCR-10-2173

[13] Sconocchia G, Zlobec I, Lugli A, Calabrese D, Iezzi G, et al (2011) Tumor infiltration by FcgammaRIII (CD16)+ myeloid cells is associated with improved survival in patients with colorectal carcinoma. Int J Cancer 128: 2663–2672. 20715106 10.1002/ijc.25609 PMC3426287

[14] Sconocchia G, Arriga R, Tornillo L, Terracciano L, Ferrone S, et al (2012) Melanoma cells inhibit NK cell functions–letter. Cancer Res 72: 5428–5429. 23047870 10.1158/0008-5472.CAN-12-1181

[15] Forssell J, Oberg A, Henriksson ML, Stenling R, Jung A, et al (2007) High macrophage infiltration along the tumor front correlates with improved survival in colon cancer. Clin Cancer Res 13: 1472–1479. 17332291 10.1158/1078-0432.CCR-06-2073

[16] Nagorsen D, Voigt S, Berg E, Stein H, Thiel E, et al (2007) Tumor-infiltrating macrophages and dendritic cells in human colorectal cancer: relation to local regulatory T cells, systemic T-cell response against tumor-associated antigens and survival. J Transl Med 5: 62. 18047662 10.1186/1479-5876-5-62 PMC2212626

[17] Pancione M, Forte N, Sabatino L, Tomaselli E, Parente D, et al (2009) Reduced beta-catenin and peroxisome proliferator-activated receptor-gamma expression levels are associated with colorectal cancer metastatic progression: correlation with tumor-associated macrophages, cyclooxygenase 2, and patient outcome. Hum Pathol 40: 714–725. 19121846 10.1016/j.humpath.2008.08.019

[18] Di CE, Forni G, Lollini P, Colombo MP, Modesti A, et al (2001) The intriguing role of polymorphonuclear neutrophils in antitumor reactions. Blood 97: 339–345. 11154206 10.1182/blood.v97.2.339

[19] Granot Z, Henke E, Comen EA, King TA, Norton L, et al (2011) Tumor entrained neutrophils inhibit seeding in the premetastatic lung. Cancer Cell 20: 300–314. 21907922 10.1016/j.ccr.2011.08.012 PMC3172582

[20] Fridlender ZG, Sun J, Kim S, Kapoor V, Cheng G, et al (2009) Polarization of tumor-associated neutrophil phenotype by TGF-beta: “N1” versus “N2” TAN. Cancer Cell 16: 183–194. 19732719 10.1016/j.ccr.2009.06.017 PMC2754404

[21] Jablonska J, Leschner S, Westphal K, Lienenklaus S, Weiss S (2010) Neutrophils responsive to endogenous IFN-beta regulate tumor angiogenesis and growth in a mouse tumor model. J Clin Invest 120: 1151–1164. 20237412 10.1172/JCI37223 PMC2846036

[22] Fridlender ZG, Albelda SM (2012) Tumor-associated neutrophils: friend or foe? Carcinogenesis 33: 949–955. 22425643 10.1093/carcin/bgs123

[23] Mantovani A (2009) The yin-yang of tumor-associated neutrophils. Cancer Cell 16: 173–174. 19732714 10.1016/j.ccr.2009.08.014

[24] Mantovani A, Cassatella MA, Costantini C, Jaillon S (2011) Neutrophils in the activation and regulation of innate and adaptive immunity. Nat Rev Immunol 11: 519–531. 21785456 10.1038/nri3024

[25] Krawisz JE, Sharon P, Stenson WF (1984) Quantitative assay for acute intestinal inflammation based on myeloperoxidase activity. Assessment of inflammation in rat and hamster models. Gastroenterology 87: 1344–1350. 6092199

[26] Heinecke JW, Li W, Francis GA, Goldstein JA (1993) Tyrosyl radical generated by myeloperoxidase catalyzes the oxidative cross-linking of proteins. J Clin Invest 91: 2866–2872. 8390491 10.1172/JCI116531 PMC443356

[27] Kanayama A, Miyamoto Y (2007) Apoptosis triggered by phagocytosis-related oxidative stress through FLIPS down-regulation and JNK activation. J Leukoc Biol 82: 1344–1352. 17709401 10.1189/jlb.0407259

[28] Roncucci L, Mora E, Mariani F, Bursi S, Pezzi A, et al (2008) Myeloperoxidase-positive cell infiltration in colorectal carcinogenesis as indicator of colorectal cancer risk. Cancer Epidemiol Biomarkers Prev 17: 2291–2297. 18768495 10.1158/1055-9965.EPI-08-0224

[29] Kerr MA, Stocks SC (1992) The role of CD15-(Le(X))-related carbohydrates in neutrophil adhesion. Histochem J 24: 811–826. 1362195 10.1007/BF01046353

[30] Sittel C, Eckel HE, Damm M, von PE, Kvasnicka HM (2000) Ki-67 (MIB1), p53, and Lewis-X (LeuM1) as prognostic factors of recurrence in T1 and T2 laryngeal carcinoma. Laryngoscope 110: 1012–1017. 10852523 10.1097/00005537-200006000-00024

[31] Mayer B, Funke I, Schraut W, Johnson JP, Schildberg FW (1998) [Expression of Lewis blood group antigens in stomach carcinoma induces metastatic potential]. Langenbecks Arch Chir Suppl Kongressbd 115: 631–634. 14518331

[32] Kadota A, Masutani M, Takei M, Horie T (1999) Evaluation of expression of CD15 and sCD15 in non-small cell lung cancer. Int J Oncol 15: 1081–1089. 10568812 10.3892/ijo.15.6.1081

[33] Hoff SD, Matsushita Y, Ota DM, Cleary KR, Yamori T, et al (1989) Increased expression of sialyl-dimeric LeX antigen in liver metastases of human colorectal carcinoma. Cancer Res 49: 6883–6888. 2573422

[34] Nakamori S, Kameyama M, Imaoka S, Furukawa H, Ishikawa O, et al (1993) Increased expression of sialyl Lewisx antigen correlates with poor survival in patients with colorectal carcinoma: clinicopathological and immunohistochemical study. Cancer Res 53: 3632–3637. 8101764

[35] Ichikawa D, Kitamura K, Tani N, Nishida S, Tsurutome H, et al (2000) Molecular detection of disseminated cancer cells in the peripheral blood and expression of sialylated antigens in colon cancers. J Surg Oncol 75: 98–102. 11064388 10.1002/1096-9098(200010)75:2<98::aid-jso5>3.0.co;2-r

[36] Sauter G, Simon R, Hillan K (2003) Tissue microarrays in drug discovery. Nat Rev Drug Discov 2: 962–972. 14654795 10.1038/nrd1254

[37] Jass JR, Atkin WS, Cuzick J, Bussey HJ, Morson BC, et al (1986) The grading of rectal cancer: historical perspectives and a multivariate analysis of 447 cases. Histopathology 10: 437–459. 3721406 10.1111/j.1365-2559.1986.tb02497.x

[38] Hampel H, Stephens JA, Pukkala E, Sankila R, Aaltonen LA, et al (2005) Cancer risk in hereditary nonpolyposis colorectal cancer syndrome: later age of onset. Gastroenterology 129: 415–421. 16083698 10.1016/j.gastro.2005.05.011

[39] Zlobec I, Minoo P, Baumhoer D, Baker K, Terracciano L, et al (2008) Multimarker phenotype predicts adverse survival in patients with lymph node-negative colorectal cancer. Cancer 112: 495–502. 18076013 10.1002/cncr.23208

[40] Zlobec I, Steele R, Terracciano L, Jass JR, Lugli A (2007) Selecting immunohistochemical cut-off scores for novel biomarkers of progression and survival in colorectal cancer. J Clin Pathol 60: 1112–1116. 17182662 10.1136/jcp.2006.044537 PMC2014838

[41] McShane LM, Altman DG, Sauerbrei W, Taube SE, Gion M, et al (2005) Reporting recommendations for tumor marker prognostic studies (REMARK). J Natl Cancer Inst 97: 1180–1184. 16106022 10.1093/jnci/dji237

[42] Gabrilovich DI, Ostrand-Rosenberg S, Bronte V (2012) Coordinated regulation of myeloid cells by tumours. Nat Rev Immunol 12: 253–268. 22437938 10.1038/nri3175 PMC3587148

[43] Condeelis J, Pollard JW (2006) Macrophages: obligate partners for tumor cell migration, invasion, and metastasis. Cell 124: 263–266. 16439202 10.1016/j.cell.2006.01.007

[44] Donskov F, von der MH (2006) Impact of immune parameters on long-term survival in metastatic renal cell carcinoma. J Clin Oncol 24: 1997–2005. 16648500 10.1200/JCO.2005.03.9594

[45] Ilie M, Hofman V, Ortholan C, Bonnetaud C, Coelle C, et al (2012) Predictive clinical outcome of the intratumoral CD66b-positive neutrophil-to-CD8-positive T-cell ratio in patients with resectable nonsmall cell lung cancer. Cancer 118: 1726–1737. 21953630 10.1002/cncr.26456

[46] Jensen HK, Donskov F, Marcussen N, Nordsmark M, Lundbeck F, et al (2009) Presence of intratumoral neutrophils is an independent prognostic factor in localized renal cell carcinoma. J Clin Oncol 27: 4709–4717. 19720929 10.1200/JCO.2008.18.9498

[47] Li YW, Qiu SJ, Fan J, Zhou J, Gao Q, et al (2011) Intratumoral neutrophils: a poor prognostic factor for hepatocellular carcinoma following resection. J Hepatol 54: 497–505. 21112656 10.1016/j.jhep.2010.07.044

[48] Biswas SK, Mantovani A (2010) Macrophage plasticity and interaction with lymphocyte subsets: cancer as a paradigm. Nat Immunol 11: 889–896. 20856220 10.1038/ni.1937

[49] Ladoire S, Martin F, Ghiringhelli F (2011) Prognostic role of FOXP3+ regulatory T cells infiltrating human carcinomas: the paradox of colorectal cancer. Cancer Immunol Immunother 60: 909–918. 21644034 10.1007/s00262-011-1046-y PMC11028605

[50] Kang JC, Chen JS, Lee CH, Chang JJ, Shieh YS (2010) Intratumoral macrophage counts correlate with tumor progression in colorectal cancer. J Surg Oncol 102: 242–248. 20740582 10.1002/jso.21617

[51] Kinouchi M, Miura K, Mizoi T, Ishida K, Fujibuchi W, et al (2011) Infiltration of CD14-positive macrophages at the invasive front indicates a favorable prognosis in colorectal cancer patients with lymph node metastasis. Hepatogastroenterology 58: 352–358. 21661395

[52] Ong SM, Tan YC, Beretta O, Jiang D, Yeap WH, et al (2011) Macrophages in human colorectal cancer are pro-inflammatory and prime T cells towards an anti-tumour type-1 inflammatory response. Eur J Immunol 42: 89–100. 22009685 10.1002/eji.201141825

[53] Rigo A, Gottardi M, Zamo A, Mauri P, Bonifacio M, et al (2010) Macrophages may promote cancer growth via a GM-CSF/HB-EGF paracrine loop that is enhanced by CXCL12. Mol Cancer 9: 273. 20946648 10.1186/1476-4598-9-273 PMC2964621

[54] Zhou Q, Peng RQ, Wu XJ, Xia Q, Hou JH, et al (2010) The density of macrophages in the invasive front is inversely correlated to liver metastasis in colon cancer. J Transl Med 8: 13. 20141634 10.1186/1479-5876-8-13 PMC2841127

[55] McLean MH, Murray GI, Stewart KN, Norrie G, Mayer C, et al (2011) The inflammatory microenvironment in colorectal neoplasia. PLoS One 6: e15366. 21249124 10.1371/journal.pone.0015366 PMC3017541

[56] Rao HL, Chen JW, Li M, Xiao YB, Fu J, et al (2012) Increased intratumoral neutrophil in colorectal carcinomas correlates closely with malignant phenotype and predicts patients’ adverse prognosis. PLoS One 7: e30806. 22295111 10.1371/journal.pone.0030806 PMC3266280

[57] Dransfield I, Buckle AM, Savill JS, McDowall A, Haslett C, et al (1994) Neutrophil apoptosis is associated with a reduction in CD16 (Fc gamma RIII) expression. J Immunol 153: 1254–1263. 8027553

[58] Ueda E, Kinoshita T, Nojima J, Inoue K, Kitani T (1989) Different membrane anchors of Fc gamma RIII (CD16) on K/NK-lymphocytes and neutrophils. Protein- vs lipid-anchor. J Immunol 143: 1274–1277. 2545784

[59] Zlobec I, Karamitopoulou E, Terracciano L, Piscuoglio S, Iezzi G, et al (2010) TIA-1 cytotoxic granule-associated RNA binding protein improves the prognostic performance of CD8 in mismatch repair-proficient colorectal cancer. PLoS One 5: e14282. 21179245 10.1371/journal.pone.0014282 PMC3003488

[60] Trellakis S, Bruderek K, Dumitru CA, Gholaman H, Gu X, et al (2011) Polymorphonuclear granulocytes in human head and neck cancer: enhanced inflammatory activity, modulation by cancer cells and expansion in advanced disease. Int J Cancer 129: 2183–2193. 21190185 10.1002/ijc.25892

[61] Theilgaard-Monch K, Jacobsen LC, Borup R, Rasmussen T, Bjerregaard MD, et al (2005) The transcriptional program of terminal granulocytic differentiation. Blood 105: 1785–1796. 15514007 10.1182/blood-2004-08-3346

[62] Hoy A, Leininger-Muller B, Kutter D, Siest G, Visvikis S (2002) Growing significance of myeloperoxidase in non-infectious diseases. Clin Chem Lab Med 40: 2–8. 11916266 10.1515/CCLM.2002.002

[63] Sihvo EI, Salminen JT, Rantanen TK, Ramo OJ, Ahotupa M, et al (2002) Oxidative stress has a role in malignant transformation in Barrett’s oesophagus. Int J Cancer 102: 551–555. 12447994 10.1002/ijc.10755

[64] Song M, Santanam N (2001) Increased myeloperoxidase and lipid peroxide-modified protein in gynecological malignancies. Antioxid Redox Signal 3: 1139–1146. 11813987 10.1089/152308601317203648

[65] Samoszuk MK, Nguyen V, Gluzman I, Pham JH (1996) Occult deposition of eosinophil peroxidase in a subset of human breast carcinomas. Am J Pathol 148: 701–706. 8774125 PMC1861714

[66] Rainis T, Maor I, Lanir A, Shnizer S, Lavy A (2007) Enhanced oxidative stress and leucocyte activation in neoplastic tissues of the colon. Dig Dis Sci 52: 526–530. 17195121 10.1007/s10620-006-9177-2

[67] Otamiri T, Sjodahl R (1989) Increased lipid peroxidation in malignant tissues of patients with colorectal cancer. Cancer 64: 422–425. 2544250 10.1002/1097-0142(19890715)64:2<422::aid-cncr2820640214>3.0.co;2-2

[68] Clark RA, Klebanoff SJ (1975) Neutrophil-mediated tumor cell cytotoxicity: role of the peroxidase system. J Exp Med 141: 1442–1447. 165258 10.1084/jem.141.6.1442 PMC2189847

[69] Colombo MP, Ferrari G, Stoppacciaro A, Parenza M, Rodolfo M, et al (1991) Granulocyte colony-stimulating factor gene transfer suppresses tumorigenicity of a murine adenocarcinoma in vivo. J Exp Med 173: 889–897. 1706752 10.1084/jem.173.4.889 PMC2190799

[70] Abi Abdallah DS, Egan CE, Butcher BA, Denkers EY (2011) Mouse neutrophils are professional antigen-presenting cells programmed to instruct Th1 and Th17 T-cell differentiation. Int Immunol 23: 317–326. 21422151 10.1093/intimm/dxr007 PMC3082529

[71] Sandilands GP, McCrae J, Hill K, Perry M, Baxter D (2006) Major histocompatibility complex class II (DR) antigen and costimulatory molecules on in vitro and in vivo activated human polymorphonuclear neutrophils. Immunology 119: 562–571. 17034427 10.1111/j.1365-2567.2006.02471.x PMC2265830

[72] Ning Y, Manegold PC, Hong YK, Zhang W, Pohl A, et al (2011) Interleukin-8 is associated with proliferation, migration, angiogenesis and chemosensitivity in vitro and in vivo in colon cancer cell line models. Int J Cancer 128: 2038–2049. 20648559 10.1002/ijc.25562 PMC3039715

[73] Trutmann M, Terracciano L, Noppen C, Kloth J, Kaspar M, et al (1998) GM-CSF gene expression and protein production in human colorectal cancer cell lines and clinical tumor specimens. Int J Cancer 77: 378–385. 9663599 10.1002/(sici)1097-0215(19980729)77:3<378::aid-ijc12>3.0.co;2-4

[74] Urdinguio RG, Fernandez AF, Moncada-Pazos A, Huidobro C, Rodriguez RM, et al (2012) Immune dependent and independent anti-tumor activity of GM-CSF aberrantly expressed by mouse and human colorectal tumors. Cancer Res 73: 395–405. 23108143 10.1158/0008-5472.CAN-12-0806

[75] Roxburgh CS, McMillan DC (2012) The role of the in situ local inflammatory response in predicting recurrence and survival in patients with primary operable colorectal cancer. Cancer Treat Rev 38: 451–466. 21945823 10.1016/j.ctrv.2011.09.001

[76] Paz-Ares LG, Gomez-Roca C, Delord JP, Cervantes A, Markman B, et al (2011) Phase I pharmacokinetic and pharmacodynamic dose-escalation study of RG7160 (GA201), the first glycoengineered monoclonal antibody against the epidermal growth factor receptor, in patients with advanced solid tumors. J Clin Oncol 29: 3783–3790. 21900113 10.1200/JCO.2011.34.8888

[77] Galon J, Franck P, Marincola FM, Angell HK, Thurin M, et al (2012) Cancer classification using the Immunoscore: a worldwide task force. J Transl Med 10: 205. 23034130 10.1186/1479-5876-10-205 PMC3554496

